# MEN2 phenotype in a family with germline heterozygous rare *RET* K666N variant

**DOI:** 10.1530/EDM-24-0009

**Published:** 2024-09-04

**Authors:** A La Greca, D Dawes, M Albuja-Cruz, C Raeburn, L Axell, L Ku, C Klein, C Marshall, L Fishbein

**Affiliations:** 1 Division of Endocrinology, Metabolism and Diabetes, Department of Medicine, University of Colorado, Aurora, Colorado, USA; 2 Internal Medicine Residency, University of Colorado, Aurora, Colorado, USA; 3 Division of GI, Trauma and Endocrine Surgery, Department of Surgery, University of Colorado, Aurora, Colorado, USA; 4 Division of Medical Oncology, Department of Medicine, University of Colorado, Aurora, Colorado, USA; 5 Department of Pathology, University of Colorado, Aurora, Colorado, USA; 6 Department of Biomedical Informatics, University of Colorado, Aurora, Colorado, USA

**Keywords:** Adult, Female, White, USA, Thyroid, Thyroid, Cardiovascular endocrinology, Genetics and mutation, Adrenal, Endocrine-related cancer, Insight into disease pathogenesis or mechanism of therapy, August, 2024

## Abstract

**Summary:**

Multiple endocrine neoplasia type 2 (MEN2) is a hereditary cancer syndrome caused by germline-activating pathogenic variants in the *RET* proto-oncogene. MEN2A is the most common subtype, with a risk for medullary thyroid cancer (MTC), pheochromocytoma (PHEO), and primary hyperparathyroidism (PHPT), whereas MEN2B is less common and associated with MTC and PHEO along with mucosal neuromas. Little is known about the specific *RET* germline heterozygous variant K666N. This variant has been described in very few families, and in most cases, patients were diagnosed with a very indolent MTC as the only feature. There is one case of MTC and bilateral PHEO. The *RET* K666N variant is not stratified yet by the American Thyroid Association, and data are limited on pathogenicity; therefore, appropriate screening and treatment of asymptomatic *RET* K666N carriers are unclear. Here, we report a family with a heterozygous germline *RET* K666N variant. The proband was identified when she experienced cardiogenic shock and multi-organ failure after an elective hysterectomy and subsequently was found to have PHEO, with genetic testing revealing the *RET* K666N germline variant. Patient consent was obtained through IRB protocol COMIRB #15-0516.

**Learning Points:**

- The specific *RET* germline heterozygous variant K666N is rare and described in very few families, and in most cases, patients were diagnosed with a very indolent MTC as the only feature. Our proband is much younger and has PHEO, MTC, and PHPT.
- The *RET* K666N germline variant appears to be a low penetrance variant for MEN2.

## Background

We present a case of a previously healthy 40-year-old female who underwent an elective partial hysterectomy complicated by decompensated Takotsubo’s cardiomyopathy and cardiogenic shock with multi-organ failure. Biochemical workup and imaging revealed a PHEO. After recovering from the complicated postoperative course, she underwent an elective retroperitoneoscopic right adrenalectomy. The pathology report confirmed a right 3.7 cm PHEO, and clinical genetic testing identified a heterozygous *RET* c.1998G>T;p.K666N germline variant (transcript NM_020975.4). She was subsequently diagnosed with medullary microcarcinoma and primary hyperparathyroidism. Most of the patients with this variant described in the literature were older at diagnosis, with MTC as the only feature. The germline *RET* K666N variant appears to be pathogenic but of low penetrance for MEN2.

## Case description

The proband was a previously healthy 40-year-old woman with dysmenorrhea and menorrhagia who underwent an elective partial hysterectomy with preservation of ovaries at an outside hospital. Her peri- and post-operative course was complicated by a hypertensive crisis, Takotsubo’s cardiomyopathy, and cardiogenic shock with multi-organ failure requiring short-term hemodialysis for acute renal failure. She then developed *Clostridium difficile* colitis resulting in a toxic megacolon requiring total colectomy and end ileostomy. This led to a need for temporary total parenteral nutrition and later enteral nutrition through a PEG tube, which was complicated by leak peritonitis. During the workup, an adrenal mass was found, and biochemical studies confirmed PHEO. For the PHEO and heart failure, she was discharged on doxazosin 4 mg twice a day and metoprolol 75 mg twice a day. After months in the hospital, she was discharged to a rehabilitation facility and eventually home with a referral to the University of Colorado Hospital Endocrinology and Endocrine Surgery for a posterior surgical approach to adrenalectomy surgery given her multiple recent abdominal surgeries.

At the Endocrinology visit, the patient recalled in retrospect several ‘spells’ over the previous 2 years that she described as a ‘surge running up towards her head’ associated with palpitations, sweating, and headaches. Family history was significant for her father having had a jaw tumor at the age of 41 years and her mother had Graves’ disease. There was no family history of PHEO or paraganglioma, renal cell carcinoma, thyroid cancer, renal stones, or sudden deaths. Her physical examination was remarkable for a cachectic appearance, an ileostomy bag, and sacral ulcer. She had no mucosal neuromas. She had a height of 1.78 m (5′10″), weight of 51.3 kg (113 lbs), BMI of 16.21 kg/m², heart rate (HR) of 137, and blood pressure (BP) of 132/87. Given her poor nutritional status and physical conditioning after the 3-month hospitalization, the decision was made to continue alpha blockade and temporarily defer surgical resection of the PHEO. A month later, once she recovered further and her nutritional status improved, she was prepped for the PHEO resection. She was continued on metoprolol. The doxazosin was switched to phenoxybenzamine to titrate up for 2 weeks prior to adrenalectomy to goal BP and HR.

**Table 1:** Genotype and phenotype characteristics of the proband and tested family members.

	Proband	Sister	Daughter	Brother	NR
*RET* germline/genotype	K666N/wt	K666N/wt	K666N/wt	K666N/wt	–
Age at diagnosis, years	40	42	21	46	–
Medullary thyroid cancer	Yes	NID	NID	NID*	–
Pheochromocytoma	Unilateral	NID	NID	NID	–
Primary hyperparathyroidism	Yes	NID	NID	NID	–
Calcitonin, ng/mL	12.3 –30.7	5.2	Normal	17.3 –22.3	0 –5.1
Plasma free metanephrine, pg/mL	1957	Normal	Normal	Normal	< 57
Plasma free normetanephrine, pg/mL	1329	Normal	Normal	Normal	< 148

*Patient has not had thyroidectomy and has mildly elevated calcitonin. No nodules on thyroid ultrasound. Cannot rule out MTC or C. NID, not identified to date; NR, normal range.

## Investigation

Her biochemical studies confirmed PHEO, with elevated plasma metanephrine of 1957 pg/mL (normal range (NR): <57 pg/mL) and plasma normetanephrine of 1329 pg/mL (NR: <148 pg/mL) (Table 1).

Her CT scan revealed a right 4.5 × 4.2 cm hypodense adrenal mass.

## Treatment

She underwent retroperitoneoscopic right adrenalectomy without complication. The pathology report confirmed a right 3.7 cm pheochromocytoma with lymphovascular invasion. Ki-67/Mib-1 was 3–4%. She was referred to the Hereditary Cancer Genetics Clinic for clinical genetic testing given her diagnosis of PHEO (1, 2, 3). Invitae 83 gene cancer panel identified a heterozygous *RET* c.1998G>T;p.K666N germline variant (transcript NM_020975.4).

In light of the *RET* variant, subsequent labs were sent and revealed an elevated PTH at 103 pg/mL (NR: 12–88), low 25-hydroxyvitamin D at 16 ng/dL, normal calcium of 9.8 mg/dL and albumin 4.1 g/dL, mildly elevated calcitonin of 12.3 pg/mL (NR: 0–5.1), with normal CEA of 1.3 ng/mL (NR: 0–3), TSH at 1.51 mIU/L, and chromogranin A at 35 ng/mL (NR: 0–95). The 8-week post-operative plasma metanephrine normalized to 0.25 nmol/mL (NR: 0–0.49 nmol/mL) and normetanephrine to 0.76 nmol/mL (NR: 0–0.89 nmol/mL). She was given vitamin D3 supplementation, and follow-up labs showed PTH at 120 pg/mL, 25-hydroxyvitamin D of 20 ng/dL, calcium of 9.8 mg/dL, calcitonin of 16.8 pg/mL, and CEA of 1.7 ng/mL. A thyroid ultrasound showed no nodules, but there was an indeterminate right-level IV cervical node. An ultrasound-guided fine-needle aspiration showed cytology that was negative for malignancy with only heterogeneous lymphoid tissue, and calcitonin and thyroglobulin needle washouts were both negative. Given the mildly elevated serum calcitonin, thyroidectomy was recommended. There was also persistent mild primary hyperparathyroidism.

## Outcome and follow-up

Given the patient’s history with a prior surgery leading to multi-organ failure, ileostomy, and prolonged recovery, she elected to monitor labs every 4–6 months rather than proceed immediately with total thyroidectomy and possible parathyroidectomy at that time. Over the course of the next 2 years, she was monitored every 6 months. Ultimately, the PTH continued to be elevated at 95 pg/mL (NR: 12–88 pg/mL), and the calcium rose to 11.4 mg/dL (8.6–10.3 ng/dL) with a low 25-hydroxyvitamin D of 13 ng/mL and a normal 24-h urine calcium of 159 mg/day. Also, the calcitonin rose to 30.7 pg/mL (NR: 0–5.1 pg/mL). These labs prompted her to agree to total thyroidectomy and partial parathyroidectomy. The left inferior parathyroid gland was removed, and pathology showed 0.1 g hypercellular tissue. The thyroid gland pathology revealed a 4 mm medullary microcarcinoma involving the left lobe of the thyroid without extrathyroidal extension. Additionally, there was a right-sided 5 mm hyalinizing trabecular tumor with negative margins. One of the 13 lymph nodes was positive for metastatic microcarcinoma with a tumor deposit of 0.2 mm. She had no complications from the surgery. Surveillance labs have continued to be normal 2 years post-operatively, with the PTH and calcium normalized to 51 pg/mL (NR: 15–88 pg/mL) and 9.3 mg/dL (NR: 8.6–10.3 mg/dL), respectively, with a 25-hydroxyvitamin D level of 33 ng/mL and a normal CEA level at 1.4 ng/mL (NR: 0–3 ng/mL) with an undetectable calcitonin (<2 pg/mL with NR: 0–5.1 pg/mL).

**Figure 1:**
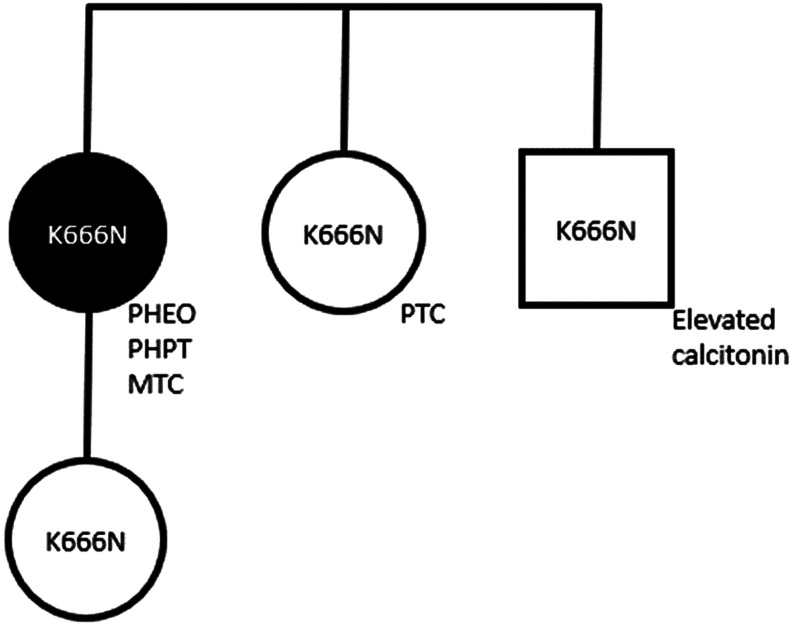
Pedigree.

**Figure 2:**
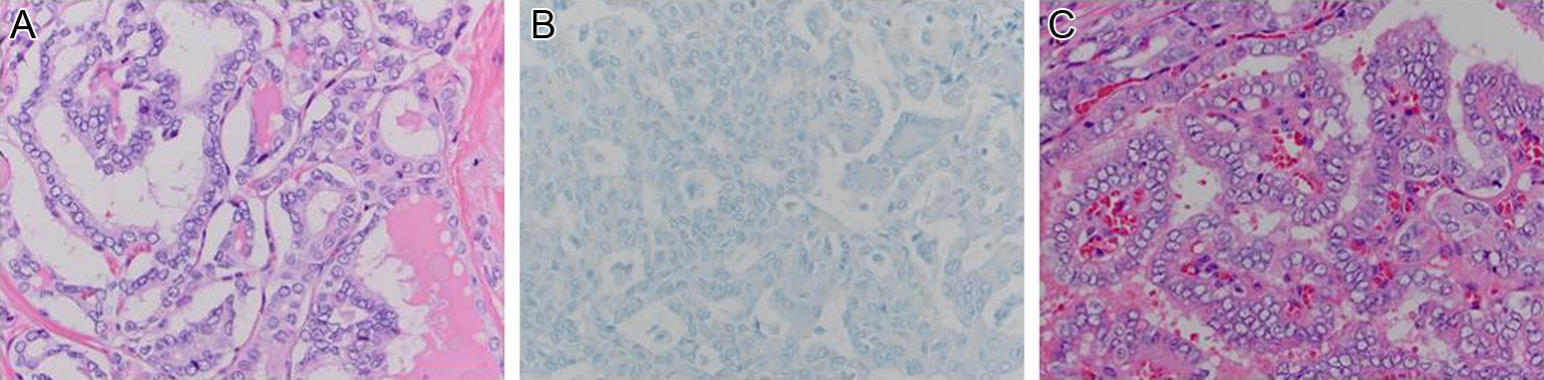
Pathology of papillary thyroid cancer. (A) Thyroid lobe with PTC on H&E stain. (B) Calcitonin immunohistochemistry was negative throughout the thyroid. (C) Metastasis in cervical lymph node of PTC on H&E stain.

Cascade family genetic testing revealed that the patient’s older sister, aged 42 years, also carried the same *RET* germline variant (Fig. 1). With this information, the sister became concerned about a neck mass she had noted a few months earlier. A thyroid ultrasound revealed two highly suspicious thyroid nodules of 5 mm and 11 mm (taller than wide, microcalcifications, ill-defined borders). In addition, there were suspicious left central neck lymph nodes, largest 12 mm, and suspicious left lateral neck level IV lymph nodes, largest 11 mm. FNA showed cytology of the 11 mm thyroid nodule and a left level IV node were positive for papillary thyroid cancer (PTC) and metastatic PTC, respectively. Her screening labs showed a serum calcitonin in the upper limit of normal (5.2 pg/mL with NR: 0–5.1 pg/mL), CEA 1.5 ng/mL (NR: 0–3 ng/mL), calcium 9.6 mg/dL (NR: 8.6–10.3 mg/dL), PTH 44 pg/mL (NR: 12–88 pg/mL), and 25-hydroxyvitamin D at 29 ng/mL. Plasma-free metanephrines and catecholamines were normal. She underwent total thyroidectomy with central and left lateral neck dissection. Surgical pathology confirmed multifocal classical PTC, greatest dimension 0.8 cm, no angioinvasion, and no extra-thyroidal extension; however, 8/47 LNs were positive for PTC, with the largest metastatic deposit 0.4 cm and no extra-nodal extension (pT1aN1b (AJCC 8th Edition)) (Fig. 2). There was no evidence of MTC or C-cell hyperplasia. Postoperative thyroglobulin was <0.1 with the presence of TgAb of 78 IU/mL by immunometric assay (NR: <4 IU/mL) and 30 IU/mL by radioimmunoassay (NR: <0.4 U/mL).

The proband’s older brother, aged 46 years, also had the *RET* variant (Fig. 1). His screening labs showed elevated calcitonin at 22.3 pg/mL (NR: 0–5.1 pg/mL). CEA, PTH, calcium, 25-hydroxyvitamin D, and plasma-free metanephrines were all within normal limits. A subsequent thyroid ultrasound did not show any nodules. Given his elevated calcitonin, the patient was counseled on the risk of MTC, and a thyroidectomy was recommended. He preferred to avoid surgery and continue with lab monitoring every 4–6 months. Over the course of 3 years, his calcitonin has remained stably elevated at 17.3 pg/mL (NR: 0–5.1 pg/mL). His CEA, PTH, calcium, hydroxyvitamin D, and metanephrines have all remained within normal limits. He has opted to continue with lab monitoring despite the risk of MTC.

Both the proband and her sister have one child each, both of whom tested positive for the variant. The proband’s daughter, aged 22, had all normal screening labs including plasma-free metanephrines, calcium, 25-hydroxyvitamin D, PTH, calcitonin, and CEA. Thyroid ultrasound was also normal. Given the low penetrance of the disease, the daughter will be followed with labs annually and elected to not undergo a total thyroidectomy at this time. The sister’s son, aged 24, lives out of state and has not yet been screened.

## Discussion

We report a case of a woman with a germline heterozygous *RET* c.1998G>T;p.K666N variant and a unilateral PHEO, medullary microcarcinoma, and PHPT. The proband’s brother, who also tested positive for the *RET* variant, has a mildly elevated calcitonin with no thyroid nodules. The proband’s daughter and sister have the same variant with no evidence of PHEO, MTC, or PHPT, although her sister has metastatic PTC.

The heterozygous *RET* K666N germline variant is considered pathogenic or likely pathogenic in ClinVar (4) and has an allele frequency of 0.00003 in gnomAD (5). Functional analysis *in vitro* showed increased kinase activity with the K666N variant (6). The codon change is in the intracellular domain exon 11, similar to FMTC variants and MEN2B (7).

The *RET* K666N variant likely has low penetrance based on the limited data available. Most of the patients described previously in the literature with this variant were in their 50s and 60s with MTC as the only feature (6, 8, 9). In one case, a 59-year-old patient with a homozygous *RET* K666N genotype had both MTC and bilateral PHEO. Our proband, in her 40s has PHEO, MTC, and PHPT. Her brother, also in his 40s, had a mildly elevated calcitonin but no thyroid nodules and has not yet had thyroidectomy. Her sister, also in her 40s, and her daughter, in her 20s, have no features of MEN2 thus far. They are all younger than those in the literature and must be monitored closely.

Taken together, the germline *RET* K666N variant appears to be pathogenic but has low penetrance for MEN2. This emphasizes the importance of describing families with this variant as more data is needed to fully understand the phenotype associated with this genotype and to fully understand penetrance. For now, our family has been counseled that this variant is likely pathogenic with low penetrance. The family members with the variant will be monitored every 6–12 months and screened for all features of MEN2.

## Declaration of interest

The authors declare that there is no conflict of interest that could be perceived as prejudicing the impartiality of the study reported.

## Funding

This work did not receive any specific grant from any funding agency in the public, commercial, or not-for-profit sector.

## Patient consent

Written informed consent for publication of clinical details and/or clinical images was obtained from the patient.

## Patient Perspective

I remember feeling symptoms during some of my high school days (1995–1996). Back then it was just an anxiety feeling I would get. I always knew when an ‘episode’ was about to start. I’d get this feeling in my feet (achy, heavy, uncomfortable feeling), and it would slowly move up my body and into my chest. It made me feel anxious and out of breath for a few minutes. In my late 20s/early 30s is when I noticed the racing heart feeling and getting lightheaded easily. If I drank any kind of alcohol, the feelings would intensify. I remember being able to stay up all night long and the better part of the next day as well without feeling overly tired. I had so much energy. At one point, I went to see my PCP because I had not been sleeping; I felt like I was on the edge and very irritable. Sometimes it felt like I was having hot flashes. The night sweats had started. There were nights that I had to change my clothes twice because I would wake up soaked. My chest hurt and I would get extremely lightheaded while taking a shower. I’d have to lay on the floor in the bathroom, so I didn’t fall down, to let the feeling pass. I would break out in what looked like hives on my legs if I took too hot of a shower or sat in a hot tub. My PCP referred me to a cardiologist. He had me wear a heart monitor for 30 days. Said it was just PVCs. My PCP then put me on anxiety medication. I took three different medications for almost 2 years. They did not help out with any of my symptoms.

About 5 years before being diagnosed with a PHEO is when the headaches started. Ninety-five percent of the time, they were in the base of my skull. Those headaches are almost indescribable. I have never felt that kind of pain before. Nothing seemed to help to ease the pain of them.

Since the removal of the PHEO (2018), I have, so far, not had any of those symptoms. I also used to get four to five UTIs within a years time, and those have stopped as well.

## Author contribution statement

ALG was involved in the patient care, evaluated the published data, drafted, and edited the manuscript. DK evaluated the published data, drafted, and edited the manuscript. MAC was involved in the patient care and editing the manuscript. CR was involved in the patient care and editing the manuscript. AL and KL were involved in the patient care, in reporting and describing the genetic variant, and in editing the manuscript. CK was involved in the patient care and editing the manuscript. CM was involved in preparing, reporting, and describing the pathology images, and in editing the manuscript. LF was involved in the patient care, evaluated the published data, and drafted and edited the manuscript.
